# The Stability and Digestive Characteristics of Soybean Protein Fibril/κ-Carrageenan Composite Gels for Riboflavin Encapsulation

**DOI:** 10.3390/foods15091491

**Published:** 2026-04-24

**Authors:** Bowen Yang, Yaqi Tang, Tianhe Xu, Shicheng Dai, Qi Fang, Guangxin Lv, Huan Wang, Lianzhou Jiang

**Affiliations:** College of Food Science, Northeast Agricultural University, Harbin 150030, China; 13946087819@163.com (B.Y.); a15009803347@163.com (Y.T.); 15830825739@163.com (T.X.); 18443602143@163.com (S.D.); fqname@163.com (Q.F.); guangxinlv2002@163.com (G.L.)

**Keywords:** soybean protein isolate, κ-carrageenan, composite gel, riboflavin, encapsulation

## Abstract

To address the environmental sensitivity and low bioavailability of riboflavin, this study constructed a soybean protein isolate fibril (SPF)/κ-carrageenan (κC) composite gel delivery system. This study systematically investigated the effects of two independent variables (protein type: SPI/SPF; κC concentration: 2, 4, 6, 8 mg/mL) on the gel structural stability, riboflavin encapsulation performance, and in vitro digestive delivery characteristics of the system. Thioflavin T (ThT) fluorescence and ultraviolet (UV) absorption spectroscopy confirmed the successful preparation of SPF and verified specific intermolecular interactions between SPF and κC. Intermolecular forces, protein leaching rates, and differential scanning calorimetry (DSC) results indicated that compared with SPI-κC composite gels, κC regulates SPF molecular conformation via hydrogen bonding and hydrophobic interactions to exert a synergistic effect. This conformational regulation significantly reduced the protein leaching rates in SPF-κC composite gels, elevated the thermal denaturation temperatures (up to 79.82 °C), and enhanced the gel structural stability. As the κC concentration increased, the environmental stability of SPF-κC riboflavin-loaded composite gels were markedly enhanced, which effectively delayed the gel degradation during simulated gastrointestinal digestion. This was manifested as a reduced protein loss rate (reduced to 22.23%). At a κC concentration of 8 mg/mL, the in vitro release mechanism of riboflavin shifted from Fickian to non-Fickian diffusion.

## 1. Introduction

Riboflavin, an isoquinoline derivative with a ribitol side chain, acts as a precursor to the coenzymes FMN (flavin mononucleotide) and FAD (flavin adenine dinucleotide). These coenzymes are involved in a wide range of biochemical reactions, encompassing electron transport, lipid metabolism, drug metabolism, and exogenous substance metabolism [1]. Riboflavin is predominantly found in dairy products, meat, and eggs. It is not capable of being endogenously synthesized by the human body; consequently, it has to be obtained from external sources. Riboflavin deficiency can exert a significant adverse impact on human health, potentially resulting in vitamin B_2_ deficiency disorders such as stomatitis and conjunctivitis [2]. As an essential vitamin, riboflavin exhibits environmental sensitivity: exposure to light and oxygen may diminish or even abrogate its biological activity. Furthermore, most riboflavin in natural foods needs to be hydrolyzed into free riboflavin in the intestines prior to being absorbed by the human body [3]. Consequently, the efficiency of riboflavin absorption in humans is relatively low. Thus, there is an urgent requirement for the development of food-grade materials capable of efficiently encapsulating riboflavin while guaranteeing human safety, which, in turn, improves its bioavailability and broadens its applications in the food industry.

Soybean protein isolate (SPI) is produced from defatted soybeans via an alkali-solubilization and acid-precipitation process [4]. As a high-quality plant-based protein, SPI possesses a balanced amino acid profile that encompasses all essential amino acids necessary for human physiological needs and is cholesterol-free, thus rendering it an ideal protein source for vegetarians and individuals with animal protein allergies [5]. However, SPI’s dense structure restricts functional properties, including its gelling ability [6]. Various methods have been developed to improve SPI’s functional characteristics, among which protein fibrillation has emerged as a prominent approach for modifying protein conformation to enhance the functional performance. Under specific low-pH and high-temperature conditions, SPI denatures and undergoes misfolding, followed by self-assembly into soybean protein fibril (SPF) [7]. During this fibrillation process, functional groups are exposed, thereby promoting interactions with various bioactive substances. Due to hydrophobic interactions, hydrogen bonds, and electrostatic interactions, SPF enables the protection and delivery of these bioactive compounds [8]. Ji et al. [9] has reported that SPF exhibits higher surface hydrophobicity than SPI, which augments its ability to form water-soluble complexes with curcumin. This confers superior antioxidant activity and sustained-release properties, thereby further enhancing curcumin’s bioaccessibility and bioavailability. Consequently, SPF is widely employed as a potent carrier for bioactive substances in functional foods.

Protein–polysaccharide composite gels are capable of enhancing the structural characteristics and functional attributes of single-protein gels while enhancing the encapsulation and transport capacity of nutrients, thereby expanding the application of SPI in functional foods. κ-Carrageenan (κC) is a linear sulfated polysaccharide exhibiting multiple functional characteristics, including gelling, thickening, and water retention [10]. Alavi et al. [11] developed whey protein aggregates/κC hydrogels as delivery carriers for curcumin, and reported that the incorporation of κC remarkably improved curcumin’s physical stability. Our research team previously successfully prepared a composite based on SPF/κC and its thermally induced gel and characterized their interactions, structure, and functional properties [12]. The results show that the SPF-κC composite gels exhibited enhanced rheological properties, improved water holding capacity, and a denser microstructure compared with SPI-κC gels. However, existing studies on SPF/κC composite systems are limited to the characterization of basic rheological and structural properties of blank gels, while their application potential as delivery carriers for photosensitive bioactive substances (represented by riboflavin) remains completely unexplored. Meanwhile, most reported SPI-polysaccharide gel delivery systems for riboflavin focus on the modification of native SPI, and the synergistic regulation of protein fibrillation and κC concentration gradient on the gel network structure, riboflavin environmental stability, and in vitro digestive release behavior has not been systematically elucidated [13,14,15]. In particular, the critical threshold of κC concentration that triggers the transition of riboflavin release mechanism in this system, which is essential for the precise design of controlled-release delivery systems, is still unclear.

To address these research gaps, we constructed a SPF/κC composite gel delivery system for riboflavin encapsulation in this study. First, the formation of SPF and the intermolecular interactions between SPI/SPF and κC were characterized via ThT fluorescence and UV absorption spectroscopy. Then, the effects of protein type (SPI/SPF) and κC concentration (2–8 mg/mL) on the structural stability of composite gels were systematically evaluated via intermolecular force analysis, protein leaching rate measurement, and differential scanning calorimetry (DSC). On this basis, we investigated the protective effect of the composite gel system on riboflavin under multiple environmental stresses (UV irradiation, thermal treatment, freeze–thaw cycles, pH fluctuations, and storage), as well as its in vitro digestive release behavior and release kinetics. This study not only expands the application of SPF/κC composite systems in the field of nutrient delivery, but also provides a theoretical basis and technical reference for the development of high-performance plant protein-based vitamin delivery systems.

## 2. Materials and Methods

### 2.1. Materials

Skimmed soybean flakes were obtained from Yihai Jiali Grain and Oil Foodstuff Co., (Qinhuangdao, China). κ-carrageenan (κC) and riboflavin (purity ≥ 98%) were purchased from Yuanye Biotechnology Co., Ltd. (Shanghai, China). Pepsin (enzyme activity ≥ 600 U/mg), trypsin (enzyme activity ≥ 600 U/mg), and a Bradford Protein Concentration Assay Kit were purchased from Solarbio Science & Technology Co., Ltd. (Beijing, China). All other chemical reagents used in the experiment were of analytical grade and purchased from domestic commercial suppliers in China.

### 2.2. Preparation of Soybean Protein Isolate (SPI), Soybean Protein Fibril (SPF), and SPI/SPF-κC Complexes and Thermogels

The preparation method of the SPI, SPF, and SPI/SPF-κC composite thermogels was based on our research team’s previous methodology reported by Fang et al. [12]. SPI was prepared via the alkali-solubilization and acid-precipitation method from skimmed soybean flakes. SPF was fabricated by adjusting the SPI solution (20 mg/mL) to pH 2.0 with 1 mol/L HCl, and heating in an oil bath at 85 °C with continuous stirring at 250 rpm for 20 h. As confirmed in our previous experiments [12], κC alone can form a self-standing gel when its concentration exceeds 8 mg/mL. SPI/SPF-κC complexes were prepared by dispersing κC powder into SPI or SPF solutions (final protein concentration: 20 mg/mL, pH 7.0) to obtain final κC concentrations of 2, 4, 6, and 8 mg/mL. The mixture was stirred at 25 °C for 2 h to ensure complete dissolution and uniform mixing of κC. The composite thermogel was prepared by placing the mixed solution in a water bath at 85 °C for 30 min for thermal gelation, followed by refrigeration at 4 °C for 12 h to complete the gel formation. The obtained SPI, SPI-κC complexes, SPF, and SPF-κC complexes and their thermogels were designated as SPI, I-2, I-4, I-6, I-8, SPF, F-2, F-4, F-6, and F-8, respectively.

### 2.3. Assay of Thioflavin T (ThT) Fluorescence

ThT was dispersed in 10 mmol/L phosphate buffer (pH 7.0, 150 mmol/L NaCl), filtered through a 0.22 μm membrane, and 50-fold diluted with the same buffer to prepare ThT working solution (0.8 mg/mL). A volume of 5 mL of working solution mixed with a 40 μL sample was incubated for 1 min, and the fluorescence intensity was quantified via a F-4500 fluorometer (Hitachi, Tokyo, Japan) at 440 nm excitation and 470–500 nm emission [16], with the background correction performed by subtracting the working solution’s fluorescence.

### 2.4. Ultraviolet (UV) Spectroscopy

Samples were diluted to a 0.2 mg/mL protein concentration with deionized water; UV absorption spectra were acquired using a UV-2550 spectrophotometer (Shimadzu, Suzhou, China) over 200–450 nm at 25 °C, with a 50 nm/min scanning speed and 5 nm slit width [17].

### 2.5. Determination of Intermolecular Forces

The method for measuring intermolecular interactions was adapted from Yang et al. [18]. A 2 g gel sample was dispersed in each of five different reagent solutions. The mixture of gel and reagent solution was fully homogenized with a high-speed homogenizer, followed by continuous stirring at room temperature for 20 min. The formulations of solutions A–E were as follows: (A) 0.05 mol/L phosphate buffer (pH 7.0); (B) buffer containing 0.086 mol/L Tris, 0.09 mol/L Gly, and 4 mmol/L Na_2_EDTA (pH 8.0); (C) Solution B supplemented with 15 mg/mL sodium dodecyl sulfate (SDS); (D) Solution C supplemented with 8 mol/L urea; (E) Solution D supplemented with 2% (*v*/*v*) β-mercaptoethanol. The treated mixtures were centrifuged at 5000× *g* for 30 min, and the protein concentration in the obtained supernatant was determined using a detergent-compatible Bradford Protein Concentration Assay Kit. The protein solubility of the sample in each reagent solution was calculated using Formula (1):

(1) $$Solubility=\frac{C_{s}}{C_{t}}\times 100\%$$

where *C_s_* and *C_t_* represent the protein concentration in the supernatant and the total protein concentration.

The difference in protein solubility between B and A represents electrostatic interactions; between C and B, hydrophobic interactions; between D and C, hydrogen bonds; and between E and D, disulfide bonds.

### 2.6. Determination of Protein Leaching Rate

The protein leaching rate was investigated following the assay method of He et al. [19]. A total of 2 g of gel samples were immersed in 8 mL of 0.05 mol/L phosphate buffer (pH 7.0) for 2 h. After immersion, the mixture was centrifuged at 3000× *g* for 10 min at 4 °C. The protein concentration in the supernatant was quantified using the Bradford Protein Concentration Assay Kit, and the protein leaching rate was calculated as the percentage of the leached protein content relative to the total protein content in the initial gel sample.

### 2.7. Determination of Differential Scanning Calorimetry (DSC)

The thermal denaturation behavior of the composite gel was measured using a differential scanning calorimeter (DSC, Mettler Toledo Corp., Zurich, Switzerland). A 5–10 mg portion of the freeze-dried composite gel powder was accurately weighed and placed in a pressure-resistant aluminum pan, and an empty aluminum pan of the same weight was used as the reference. The sealed pan was heated from 20 to 180 °C at a heating rate of 10 °C/min, with a nitrogen flow rate of 20 mL/min throughout the measurement. To evaluate the thermal properties of the sample in its original prepared state, no first heating cycle was performed to eliminate the thermal history of the sample. The denaturation temperature (Td) of the sample was obtained from the DSC thermogram [20].

### 2.8. Preparation of Riboflavin-Loaded Gel

Riboflavin was accurately weighed and dispersed in deionized water to prepare a 1.0 mg/mL riboflavin stock solution, which was stored in the dark at 4 °C for later use. The riboflavin stock solution was added to the SPI/SPF-κC complex solutions prepared in Section 2.2, with a final riboflavin concentration of 0.1 mg/mL in the mixed system. The mixture was stirred at 25 °C for 2 h in the dark to ensure the uniform dispersion of riboflavin in the protein–polysaccharide system. Riboflavin-loaded composite gels were prepared according to the procedure described in Section 2.2. As confirmed in our previous work [12], SPI, SPF (κC = 0 mg/mL), and I-2 (κC = 2 mg/mL) failed to form thermally induced gels with mechanical stability, and thus, the final riboflavin-loaded gel samples were designated as I-4, I-6, I-8, F-2, F-4, F-6, and F-8, consistent with the nomenclature established in Section 2.2. All operations were performed in the dark to avoid photodegradation of the riboflavin.

### 2.9. Stability Assessment of Composite Gel Delivery Systems Under Different Environmental Conditions

#### 2.9.1. Determination of UV Stability in Composite Gel Delivery Systems

A total of 10 g of riboflavin-loaded composite gel with a thickness of 1 cm was placed in a 25 mL beaker and stored under ultraviolet light at 25 °C for 5 h. The UV stability of riboflavin in the composite gel was evaluated by measuring the residual riboflavin content in the gel after irradiation.

Determination of riboflavin content: 30 mg of hydrogel lyophilized powder containing riboflavin was dissolved and centrifuged at 10,000× *g* for 15 min. The absorbance of the supernatant at a 445 nm wavelength was determined after deducting the background of the unloaded riboflavin gel [21,22,23,24].

#### 2.9.2. Determination of Thermal Stability in Composite Gel Delivery Systems

A total of 10 g of riboflavin-loaded composite gel was placed in a 25 mL beaker and stored at 4, 25, 35, 65, and 100 °C for 2 h. The thermal stability of riboflavin in the composite gel was characterized using the residual rate of riboflavin after treatment at different temperatures; the riboflavin content was determined as described in Section 2.9.1.

#### 2.9.3. Determination of Freeze–Thaw Stability in Composite Gel Delivery Systems

A total of 10 g of riboflavin-loaded composite gel was placed in a 25 mL beaker and stored at −20 °C for 12 h to be frozen. Then, it was thawed at room temperature for 5 h until completely melted. Three freeze–thaw cycles were performed, and the freeze–thaw stability of riboflavin in the riboflavin-loaded composite gel was characterized by measuring the riboflavin content in the composite gel after each cycle; the riboflavin content was determined as described in Section 2.9.1.

#### 2.9.4. Determination of Relative pH Stability in Composite Gel Delivery Systems

A total of 10 mL of PBS (0.1 mmol/L) solutions at different pH values (2, 4.5, 7, 9.5, 12) was added to 10 g of the riboflavin-loaded composite gel. After 2 h standing at room temperature, the riboflavin content in the composite gel was determined. This value represents the relative pH stability of riboflavin in the riboflavin-loaded composite gel; the riboflavin content was determined as described in Section 2.9.1.

#### 2.9.5. Determination of Storage Stability of Composite Gel Delivery Systems

A total of 10 g of riboflavin-loaded gel was placed in a 25 mL beaker and stored at room temperature, where it was protected from light for 7 days. The storage stability of riboflavin in the composite gel was characterized using the riboflavin content in the riboflavin-loaded composite gel; the riboflavin content was determined as described in Section 2.9.1.

### 2.10. Determination of Protein Loss Rate in Composite Gel Delivery Systems

Simulated gastric fluid (SGF) and simulated intestinal fluid (SIF) without enzymes were prepared according to the standards of the United States Pharmacopeia (2004 edition) [25]. The SGF was prepared as follows: 2.0 g of NaCl and 7 mL of 37% concentrated hydrochloric acid were dissolved in 800 mL of deionized water, the pH of the solution was adjusted to 1.2 with 1 mol/L HCl, and the volume was adjusted to 1 L with deionized water. Enzyme-free SIF was prepared as follows: 6.8 g KH_2_PO_4_ was dissolved in 250 mL deionized water, 190 mL 0.2 mol/L NaOH was added, the pH was adjusted to 7.5 with 0.2 mol/L NaOH, and the mixture was diluted to 1 L with deionized water.

The protein loss rate of the composite gel in simulated digestive fluids was determined according to the method reported by Lian et al. [26]. The composite gel sample was freeze-dried for 48 h, and an appropriate amount of lyophilized gel powder was accurately weighed and added to the enzyme-free SGF or SIF. The mixture was incubated at 37 °C for 3 h, and an aliquot of the solution was collected every 30 min during the incubation. The collected solution was centrifuged at 1000× *g* for 10 min at 4 °C, and the protein concentration in the supernatant was quantified using the Bradford Protein Concentration Assay Kit. The protein loss rate was calculated using Formula (2):

(2) $$Protein\;loss\;rate\left(\%\right)=\frac{Protein\;content\;released\;at\;(t)\;time}{Total\;Protein\;Content\;}\times 100\%$$

### 2.11. In Vitro Digestion and Release Kinetics

Gastric phase: The sample (5 mL) was mixed with simulated gastric fluid (15 mL). Subsequently, the mixture’s pH was adjusted to 2.0 using HCl or NaOH, and the mixture was shaken for 2 h at 100 rpm and 37 °C using a thermostatic water bath shaker.

Small intestinal stage: The 20 mL gastric-stage solution pH was adjusted to 7.0, mixed with simulated intestinal fluid at 1:1 (*v*/*v*), maintained at pH 7.0 via dropwise 0.1 mol/L NaOH, and shaken for 2 h at 37 °C and 100 rpm in a thermostatic water bath shaker.

Riboflavin content measurement: 5 mL of the digested solution was centrifuged (1000× *g*, 5 min) and the supernatant was harvested; the absorbance was quantified at 445 nm, and the content was calculated based on the standard curve.

The release kinetic study involved fitting using the Ritger–Peppas model Formula (3):

(3) $$ln\left(\frac{M_{t}}{M_{\infty }}\right)=ln\left(k\right)+n\times ln(t)$$

In the equation, *M_t_*/*M*_∞_ is the drug release fraction at time *t*, *k* is the release rate constant, *n* is the release exponent, and *t* is the elapsed time.

### 2.12. Statistical Analysis

All experiments were run in triplicate or more; data analysis was performed via SPSS 24.0 (SPSS Inc., Evanston, IL, USA). The results are presented as the mean ± standard error, with statistical significance set at *p* < 0.05.

## 3. Results and Discussion

### 3.1. Analysis of Thioflavin T (ThT) Fluorescence in Complexes

**Figure 1:**
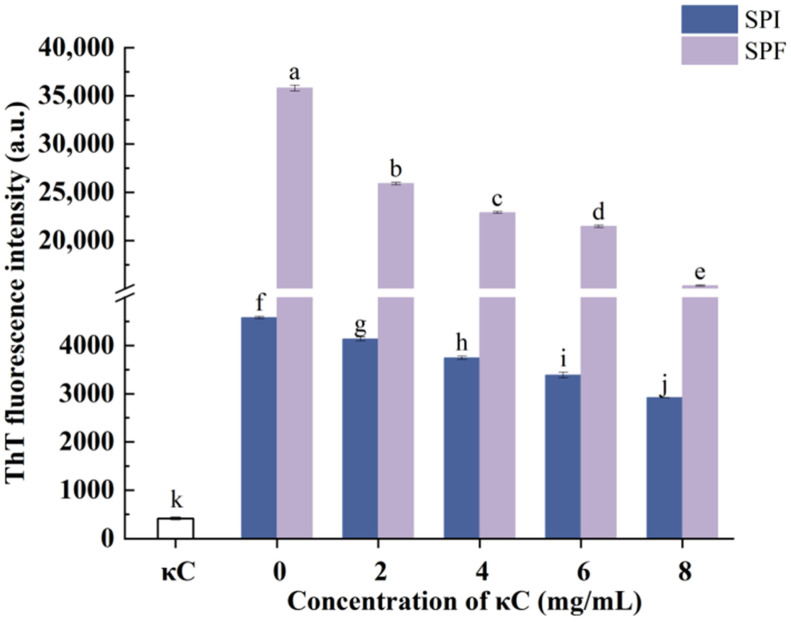
ThT fluorescence intensity of κC and SPI-κC/SPF-κC complex at different κC concentrations. The white legend indicates κC solution without SPI and SPF addition. Different letters (a–k) indicate significant differences between sample means (*p* < 0.05).

ThT is a small molecule substance that exhibits intense fluorescence upon binding to protein fibrils. ThT is widely used as a probe for detecting protein fibril formation [27]. Figure 1 shows the ThT fluorescence intensity of SPI/SPF-κC complexes with varying κC concentrations. The binding of SPF to ThT yields a higher fluorescence intensity than that of SPI, which is attributed the conformational transition of SPI during the fibrillation process. During the acid–heat induced fibrillation, SPI unfolds and self-assembles into fibril structures rich in cross β-sheet conformation, which provides abundant specific binding sites for ThT molecules. These binding sites effectively restrict the rotational motion of the ThT excited state, thus boosting the fluorescence quantum yield and markedly elevating the fluorescence intensity [28]. The significant increase in ThT fluorescence intensity of SPF (*p* < 0.05) further verifies the successful formation of SPF [29]. After the incorporation of κC, the ThT fluorescence intensity of SPI-κC/SPF-κC complexes declined with rising κC concentration, consistent with Feng et al.’s findings [20]. This reduction may be attributed to the addition of polysaccharides limiting the specific binding of ThT molecules to the fibers, resulting in decreased fluorescence intensity.

### 3.2. Analysis of the Ultraviolet (UV) Spectrum of the Complex

**Figure 2:**
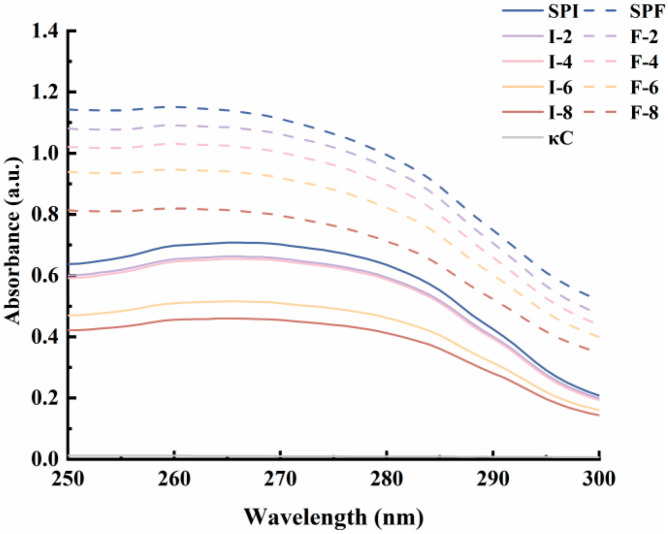
UV spectra of κC, SPI/SPF, and SPI-κC/SPF-κC complexes with different κC concentrations.

Ultraviolet spectroscopy is a widely employed technique for investigating interactions between proteins and other molecules [30]. The UV spectra of SPI, SPF, and the SPI-κC and SPF-κC complexes are shown in Figure 2. The maximum absorption peak for all samples appears at approximately 270–280 nm, a phenomenon ascribed to the absorption of conjugated double bonds in tryptophan and tyrosine residues within the proteins at this specific wavelength [31]. Compared with SPI, SPF exhibited an enhanced UV absorption peak intensity. This observation is attributed to the unfolding of protein structures, elongation of peptide chains, and increased exposure of aromatic amino acids to the solvent upon acid–heat treatment [32]. With rising κC concentration, the UV absorption peak intensities of SPI and SPF declined progressively. Possible reasons include the following: (1) κC primarily absorbs in the UV region through its carbonyl (C=O) and carboxyl groups (-COOH), while lacking aromatic structures that contribute significantly to UV absorption; (2) the incorporation of κC may inhibit the exposure of tryptophan and tyrosine residues, thereby reducing the UV absorption peak intensities of SPI and SPF; (3) the formation of complexes between SPI/SPF and κC may induce alterations in the spatial conformations of the proteins, which, in turn, influences changes in their UV absorption spectra [33].

### 3.3. Analysis of Intermolecular Forces in Composite Gels

**Figure 3:**
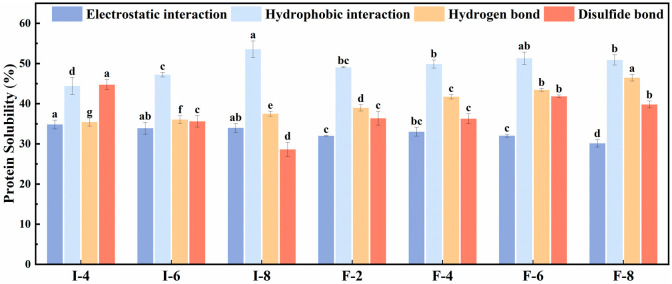
Intermolecular forces of SPI-κC composite gels and SPF-κC composite gels prepared at different κC concentrations. Different letters (a–g) indicate significant differences between sample means (*p* < 0.05).

Analysis of the solubility of composite gels in different solvents enables the identification of the major intermolecular forces involved in composite gels. These intermolecular interactions play a pivotal role in the formation and preservation of the structural stability of composite gels [34]. In the present study, various reagents were used to explore the underlying interactions between SPI/SPF and κC in the gel network. Given that different solvents can disrupt specific chemical bonds, higher protein solubility in a particular solvent reflects enhanced dispersion of the composite gel in that system, indicating a more significant contribution of the disrupted intermolecular force to gel structure. Figure 3 illustrates that the intermolecular forces in SPI-κC and SPF-κC composite gels display subtle variations with increasing κC concentration. SPF-κC composite gels show a increase in hydrogen bonds. This observation is associated with the unique structure of SPF, which is rich in β-sheet conformations—formations that are hydrogen-bond-dependent [35]. With increasing κC concentration, hydrogen bond and hydrophobic interactions in the SPI-κC/SPF-κC composite gels are significantly enhanced. κC is rich in hydrophilic groups (e.g., hydroxyl groups) that can form hydrogen bonds with free water molecules within the gel matrix [36]. The enhanced hydrophobic interaction in SPI-κC/SPF-κC composite gels may be attributed to two primary mechanisms: (1) heating-induced thermal denaturation of proteins, which exposes internal hydrophobic sites, and (2) interactions between SPI/SPF and κC, which promote the exposure of protein hydrophobic groups, thereby strengthening the hydrophobic interaction within the composite gel [37]. Electrostatic interaction also play a role in the formation of SPI-κC/SPF-κC composite gels. With increasing κC concentration, electrostatic interaction in the composite gels remain relatively stable or slightly diminish. This may be due to fact that the overall negative charge density of the system increases significantly with the increase of κ-carrageenan concentration, and the long-range electrostatic repulsion between protein and polysaccharide molecules is enhanced [38]. This repulsion reduces the effective collision and binding probability between protein and polysaccharide molecules, further weakening the formation of local electrostatic attraction complexes and causing the contribution of electrostatic interaction to the gel network to remain stable or slightly decrease [38]. Disulfide bonds are covalent crosslinks formed by the oxidation of exposed sulfhydryl groups, exerting a critical influence on the structure of composite gels. Low κC concentrations facilitate the formation of disulfide bonds in SPI/SPF, suggesting that the addition of κC activates buried disulfide bonds and induces the formation of new intermolecular covalent bonds [39]. At optimal κC concentrations, κC molecules bind to free water in the composite gel, which enhances interprotein disulfide bond formation and improves the integrity of the gel network structure [40]. As the κC concentration increases further, the disulfide bond content in the SPI-κC/SPF-κC composite gels decreases to varying extents. This decrease may be due to the increased viscosity of the system, which reduces the exposure of sulfhydryl groups in proteins.

### 3.4. Analysis of Protein Leaching Rate in Composite Gels

**Figure 4:**
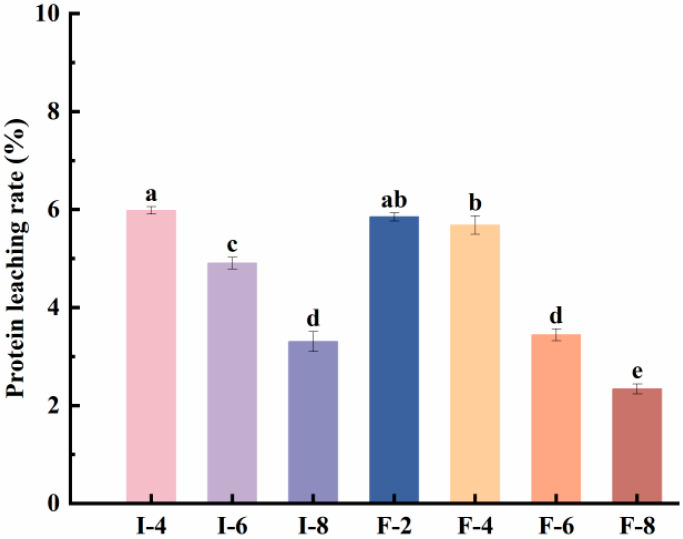
Protein leaching rate of SPI-κC/SPF-κC composite gels prepared at different κC concentrations. Different letters (a–e) indicate significant differences between sample means (*p* < 0.05).

The protein leaching rate is defined as the percentage of protein that dissolves into the phosphate buffer (0.05 M, pH 7.0) when the gel is immersed in the buffer. This rate is correlated with interactions within the gel matrix, and excessive protein elution is recognized as an unfavorable property of gels [19]. Figure 4 presents the protein elution rates of seven gel samples. The results demonstrate that with a rising κC concentration, the protein elution rate of the SPI-κC/SPF-κC composite gel samples gradually decreased. This implies that hydrogen bond and hydrophobic interactions between κC and SPI/SPF enhance the structural properties of the composite gel and markedly improve its ability to encapsulate proteins. At identical κC concentrations, the protein elution rate of the SPF-κC composite gels was lower than that of SPI-κC composite gels. This suggests that after the acid–heat treatment, SPI forms SPF with a larger effective surface area, thereby providing more binding sites for κC. The stronger interactions between SPF and κC confer greater stability to the composite gels and a lower protein leaching rate.

### 3.5. Analysis of Composite Gels by Differential Scanning Calorimetry (DSC)

**Figure 5:**
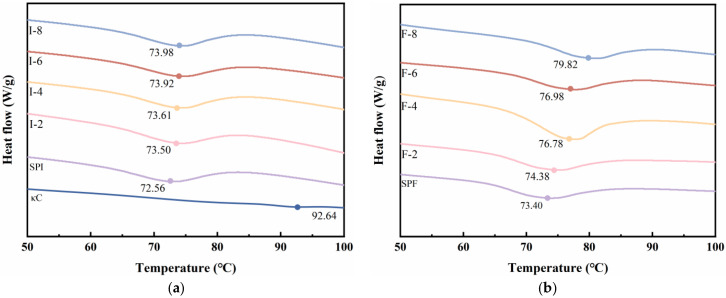
DSC of (**a**) SPI-κC composite gels and (**b**) SPF-κC composite gels prepared at different κC concentrations.

DSC is a robust technique that was used to evaluate thermal stability of SPI-κC/SPF-κC composite gels, which reflects the protein denaturation degree within the gels. Figure 5 presents the DSC profiles of all composite gel samples, with denaturation temperatures of 72.56, 73.40, and 92.64 °C for SPI, SPF, and κC, respectively. Previous studies have reported the denaturation temperature of SPI as approximately 75 °C, primarily owing to β-conglycinin (the major component of SPI) exhibiting a denaturation temperature of 75 °C [40]. The protein content of SPI also affects its denaturation temperature. Compared with SPI, SPF displays a higher denaturation temperature, which confirms that SPI’s thermal stability is enhanced following fibrillation. Dai et al. [41] similarly observed improved thermal stability in collagen after fibrillation. With increasing κC concentration, the denaturation temperature of SPI-κC/SPF-κC composite gels gradually increased, where the SPF-κC composites exhibited more significant changes. This phenomenon may be attributed to the improved thermal tolerance of composite gels arising from the interactions and cross-linking between polysaccharide and protein components. Compared with SPI, SPF possesses more binding sites for κC and forms stronger interactions with it. This observation aligns with Liu et al.’s findings [42], who demonstrated that xanthan gum forms a protective layer around SPI, thereby inhibiting or suppressing its thermal denaturation. The DSC results are consistent with the hardness test results, further confirming that elevated κC concentrations facilitate the formation of a stable gel network structure, thereby enhancing thermal stability. At 8 mg/mL κC, the SPF-κC composite gel exhibits the highest thermal resistance.

### 3.6. Analysis of the Stability of Composite Gel Delivery Systems Under Different Environmental Conditions

#### 3.6.1. Analysis of the UV Stability of Composite Gel Delivery Systems

**Figure 6:**
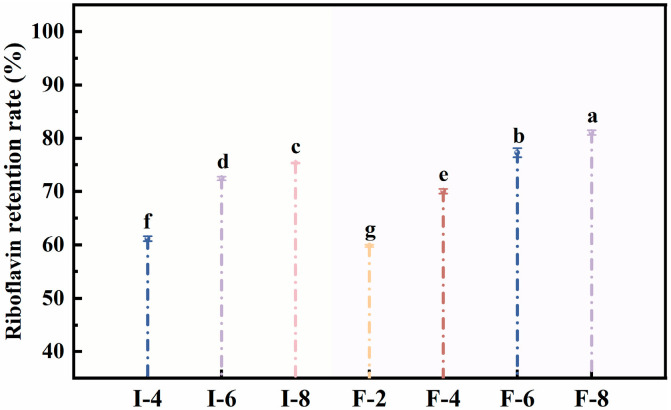
UV stability of SPI-κC/SPF-κC riboflavin-loaded composite gels prepared at different κC concentrations. Different letters (a–g) indicate significant differences between sample means (*p* < 0.05).

All riboflavin-loaded carriers were irradiated with ultraviolet (UV) light for 5 h, and the riboflavin content in the gels was measured to assess the encapsulation efficiency of the composite gel systems. Under UV irradiation, the riboflavin content decreases in all the composite gel carriers [23]. Figure 6 shows that the riboflavin retention rate of SPI-κC/SPF-κC composite gels gradually increased with rising κC concentration. At a κC concentration of 8 mg/mL, the SPI-κC composite gel achieved a riboflavin retention rate of 75.35% ± 0.12%, representing a 22.27% ± 0.15% increase compared with the lower concentrations of SPI-κC composite gels. In contrast, the SPF-κC composite gel delivery system exhibited a retention rate of 81.05% ± 0.46%, which was a 21.28% ± 0.19% increase relative to the lower concentrations of SPF-κC composite gels. This indicates that κC incorporation facilitates the formation of a dense gel network, providing effective protection for riboflavin against UV-induced degradation [43]. At the same κC level, the riboflavin retention rate of the SPF-κC composite gel was markedly higher than that of the SPI-κC composite gel (*p* < 0.05). This may be attributed to the stronger internal interactions within SPF, which form a more robust and stable gel network structure capable of encapsulating greater amounts of riboflavin and shielding it from UV damage. Overall, κC incorporation and protein fibrillation enhance the photostability of the delivery systems. Li et al. [38] has similarly reported that a higher gel density endows delivery carriers with greater UV resistance, thereby strengthening the protection of bioactive compounds under harsh environmental conditions.

#### 3.6.2. Analysis of the Thermal Stability of Composite Gel Delivery Systems

**Figure 7:**
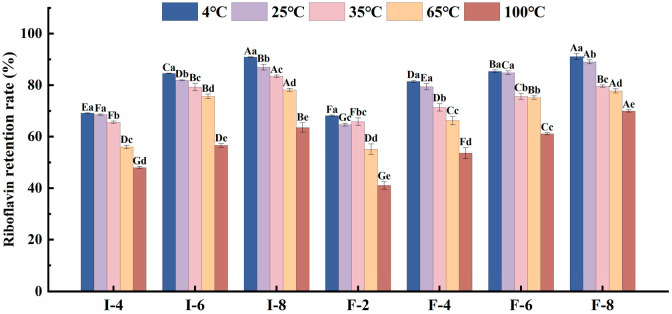
Thermal stability of SPI-κC/SPF-κC riboflavin-loaded composite gels prepared at different κC concentrations. Different uppercase letters (A–G) indicate significant differences between groups (*p* < 0.05), while different lowercase letters (a–e) indicate significant differences within groups (*p* < 0.05).

The thermal stability of the composite gels was assessed via determining the riboflavin retention rate at different temperatures, including 4 °C (refrigeration temperature), 25 °C (room temperature), 35 °C (summer high temperature), 65 °C (control), and 100 °C (control). Figure 7 presents the riboflavin retention rates of SPI-κC/SPF-κC composite gels at various temperatures. Seven composite gel samples maintained retention rates that exceeded 65%. This indicates that the composite gel carriers possess considerable environmental adaptability and thermal stability. However, at temperatures above 65 °C, the riboflavin retention rates of all composite gel delivery carriers decreased significantly (*p* < 0.05). This phenomenon might stem from thermal-processing-induced changes in hydrogen bonds, hydrophobic interactions, and spatial conformation of proteins, which, in turn, disrupt the internal intermolecular forces of the composite gels [44]. Under high-temperature conditions (65 °C and above), the riboflavin retention rates of all composite gel delivery systems increased with increasing κC concentration. At a κC concentration of 8 mg/mL, the SPI-κC and SPF-κC composite gel delivery systems achieved riboflavin retention rates of 78.19% ± 0.49% (65 °C) and 63.23% ± 1.63% (100 °C), and 77.64% ± 0.63% (65 °C) and 69.93% ± 0.36% (100 °C), respectively. This may be explained by κC enhancing the protective capacity of the delivery system for bioactive substances: an increased polysaccharide concentration strengthens the gelation effect of the system, restricting the mobility of water molecules within the gel during heating and thereby improving the protective efficiency of the composite gel delivery system for riboflavin [12]. At the same κC concentration, the SPF-κC composite gel delivery system exhibited higher riboflavin retention rates. This is probably because protein fibrillation promotes the formation of a denser gel network structure, which acts as a robust physical barrier to limit the heat transfer and inhibit the interaction between oxygen and riboflavin during heating, thereby enhancing the thermal stability of riboflavin. Dai et al. [45] has similarly reported that denser gel networks provide superior protection for encapsulated β-carotene against photo- and thermal-induced degradation.

From the perspective of practical food processing, the thermal and photosensitivity of riboflavin has long been the key limitation for its application in thermally processed foods. The thermal stability of riboflavin achieved by the SPF-κC composite gel system can fully meet the requirements of mainstream thermal processing technologies in the food industry. For conventional low-temperature pasteurization (60–65 °C, 30 min) and high-temperature short-time pasteurization (72–75 °C, 15 s) widely used in beverage and dairy industries, the riboflavin retention rate of the F-8 group reached 77.64% ± 0.63% after treatment at 65 °C for 2 h, which is far higher than the actual heat load of pasteurization, indicating that the system can achieve almost no loss of riboflavin during conventional pasteurization [46]. For commercial sterilization of canned and gel foods (121 °C, 15–20 min), the heat load of 100 °C treatment for 2 h in this study is significantly higher than that of conventional sterilization processes, while the F-8 group still maintained a riboflavin retention rate of 69.93% ± 0.36% under this harsh condition, confirming that the system has sufficient stability to resist the thermal impact of commercial sterilization.

#### 3.6.3. Analysis of Freeze–Thaw Stability in Composite Gel Delivery Systems

**Figure 8:**
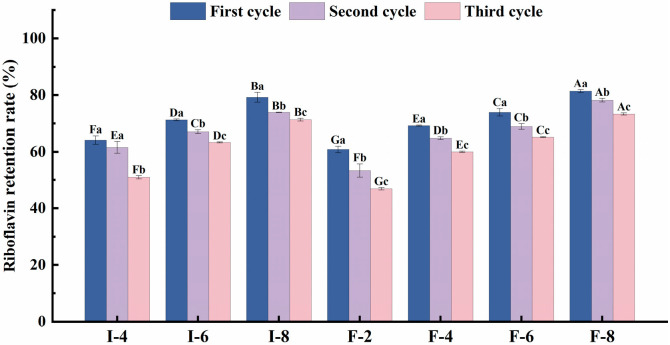
Freeze–thaw stability of SPI-κC/SPF-κC riboflavin-loaded composite gels prepared at different κC concentrations. Different uppercase letters (A–G) indicate significant differences between groups (*p* < 0.05), while different lowercase letters (a–c) indicate significant differences within groups (*p* < 0.05).

Freeze–thaw stability is a key property of gel-based foods, indicating a composite gel’s capacity to resist structural damage during freeze–thaw cycles (FTCs). Composite gels subjected to FTCs experience ice crystal volume expansion, resulting in irreversible damage to food texture, appearance, and flavor [47], compromising the sensory quality and shelf life. This limits the application of composite gel delivery systems in frozen foods and impairs the riboflavin stability. After all the riboflavin-loaded carriers were subjected to three FTC cycles, the riboflavin retention rates are presented in Figure 8; the riboflavin retention rates of all the carriers decreased progressively with an increasing number of FTC cycles. This phenomenon is primarily attributed to the following mechanisms: during gel freezing, low temperatures trigger water crystallization, and enhanced protein concentration in the gel system causes ice crystal melting, phase separation between the gel matrix and water, and gel dehydration shrinkage as the temperature rises, thereby disrupting the gel network structure [48]. Furthermore, the substantial expansion pressure generated during FTCs additionally compromises the integrity of the gel network [49]. After three FTC cycles, the riboflavin retention rate of the SPI-κC/SPF-κC composite gel carriers gradually increased with increasing κC concentration. At identical κC concentrations, the SPF-κC composite gel delivery system suffered less structural damage from FTCs than the SPI-κC counterpart, further confirming that a denser gel network structure enhances the freeze–thaw resistance and improves the riboflavin stability. Cui et al. [50] also confirmed that glycosylation can strengthen the gel structure and functional properties, with an optimal freeze–thaw resistance achieved when the gel structure is in its most compact and stable state.

#### 3.6.4. Analysis of Relative pH Stability in Composite Gel Delivery Systems

**Figure 9:**
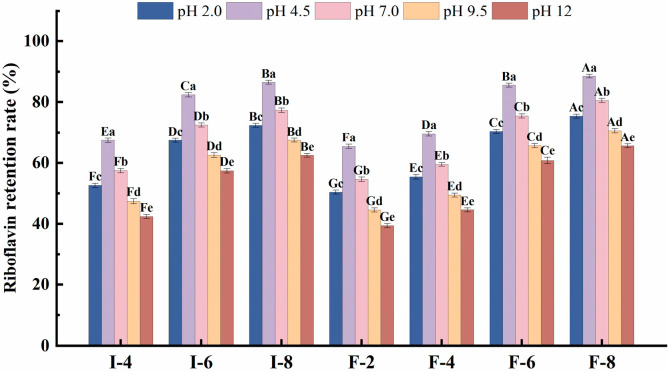
pH stability of SPI-κC/SPF-κC riboflavin-loaded composite gels prepared at different κC concentrations. Different uppercase letters (A–G) indicate significant differences between groups (*p* < 0.05). Different lowercase letters (a–e) indicate significant differences within groups (*p* < 0.05).

The stability of nutrients in food is pH-dependent. Evaluating the relative pH stability of composite gel delivery systems is crucial for protecting nutrients from degradation, minimizing losses during processing and storage, and achieving targeted nutrient release. In this study, the riboflavin retention rates of the composite gel systems at pH 2.0 (extremely acidic), pH 4.5 (protein isoelectric point), pH 7.0 (neutral), pH 9.5 (alkaline), and pH 12.0 (extremely alkaline) are shown in Figure 9. At pH 4.5, all carrier systems exhibited the highest riboflavin retention rates compared with the other pH values. This phenomenon is attributed to two primary mechanisms: First, riboflavin degradation is associated with its redox behavior, and the redox potential of riboflavin is pH-dependent. This degradation process involves the oxidation of the ribose side chain in riboflavin molecules. The redox potential reaches its minimum at pH 5.0, resulting in the slowest riboflavin redox rate [51]. Thus, riboflavin exhibits maximum stability near pH 5.0. Second, when the pH nears the protein’s isoelectric point, interactions between protein molecules are strengthened [52], which further improves the riboflavin stability. At high κC concentrations (>6 mg/mL), the riboflavin retention rate of the SPI-κC/SPF-κC composite gel delivery system remained above 50%, indicating the system’s robustness against pH variations. At the same pH, riboflavin retention rates increased with increasing κC concentrations in both systems. At identical κC concentrations, the SPF-κC composite gel delivery system exhibited higher riboflavin retention than the SPI-κC system. This further confirms that the incorporation of κC and protein fibrillation effectively reinforces the protein gel structure, forming a more stable protective barrier for riboflavin that inhibits its degradation in harsh pH environments and enhances the relative pH stability of the composite gel delivery system.

#### 3.6.5. Analysis of Storage Stability of Composite Gel Delivery Systems

**Figure 10:**
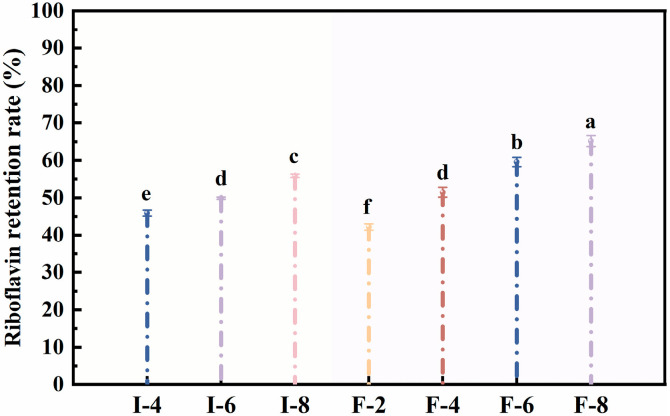
Storage stability of SPI-κC/SPF-κC riboflavin-loaded composite gels prepared at different κC concentrations. Different letters (a–f) indicate significant differences between sample means (*p* < 0.05).

Evaluating the storage stability of composite gel delivery systems is essential for maintaining product quality throughout the shelf life and preventing nutrient loss or sensory deterioration caused by gel degradation or protein denaturation. The storage stability was assessed by measuring the riboflavin retention in the composite gel delivery systems following 7 days of storage at room temperature. Figure 10 shows that as the concentration of κC increases, the retention rate of riboflavin in the SPI-κC/SPF-κC composite gel delivery system rises. This is because the incorporation of κC promotes a more robust gel network structure, reducing oxidative damage to the composite gel and thereby minimizing water evaporation within it. This alleviates the degradation of the gel network, effectively reducing the riboflavin loss [53]. Huang et al. [54] has also reported that increasing the κC concentration enhances the gel network strength, inhibits the diffusion of oxidants and pro-oxidants, and reduces the oxidative damage to emulsion gels. At identical κC concentrations, the riboflavin retention rate of the SPF-κC composite gel surpasses that of the SPI-κC composite gel. This confirms that fibrillation-treated proteins undergo structural unfolding, which creates additional binding sites for riboflavin and consequently improves its retention rate.

### 3.7. Analysis of Protein Loss Rate in Composite Gel Delivery Systems

**Figure 11:**
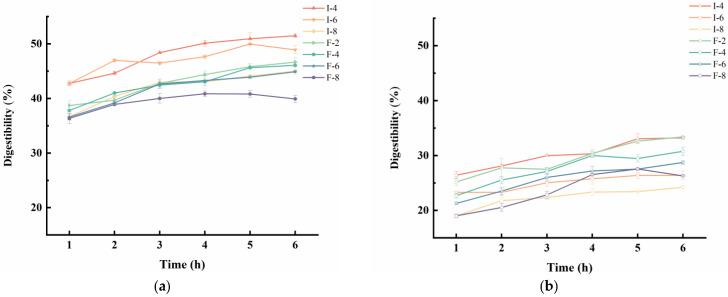
Protein loss rate of SPI-κC/SPF-κC riboflavin-loaded composite gels prepared at different concentrations of κC in (**a**) gastric phase and (**b**) intestinal phase.

Determination of the protein loss rate of composite gel systems in the gastric and intestinal phases enables the evaluation of their stability and functional maintenance capacity in distinct digestive tract environments [44]. Figure 11 shows the protein loss rates of all samples in simulated gastric fluid (SGF) and simulated intestinal fluid (SIF) without digestive enzymes. Overall, after 6 h of incubation, the protein loss rate of all samples in SGF was approximately 10% lower than that in SIF. This reduction may be attributed to the fact that the protein loss rate of protein gels is influenced by the polymer pH and ionic bond interactions, with the protein solubility being lower under acidic conditions than under neutral conditions [55]. In the SGF and SIF, the protein loss rates of the SPI-κC/SPF-κC composite gel delivery systems gradually decreased with increasing κC concentration, indicating that κC effectively inhibits gel disintegration and reduces protein leakage. Under identical experimental conditions and κC concentrations, the protein loss rate of the SPF-κC composite gel delivery system was lower than that of the SPI-κC system. This is because protein fibrillation induces structural unfolding, thereby increasing the effective surface area of the protein. Liu et al. [56] has reported that enhancing the total surface area of emulsion gels improves their in vitro digestive stability. Overall, the incorporation of κC and application of protein fibrillation effectively enhance the digestive stability of protein gels, thereby reducing the protein loss in simulated gastrointestinal fluids.

### 3.8. Analysis of Release Kinetics in Composite Gel Delivery Systems

**Figure 12:**
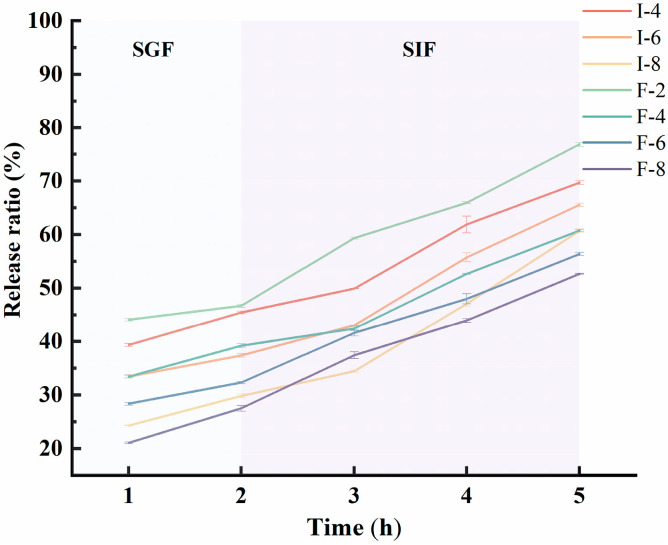
Release behavior of riboflavin in continuous gastrointestinal fluids by SPI-κC/SPF-κC riboflavin-loaded composite gels prepared at different concentrations of κC.

The release behavior of riboflavin from composite gels is largely determined by the stability of the gel network structure. Figure 12 shows the in vitro riboflavin release curve analysis based on simulated gastric and intestinal digestion. As the κC concentration increased, the riboflavin release rate of SPI-κC/SPF-κC composite gels gradually decreased, particularly when the κC concentration exceeded 6 mg/mL. This stability arises from the protective intermolecular interactions between SPI/SPF and κC, which shield the composite gel framework from degradation by pepsin and trypsin [57]. Under identical experimental conditions, the release rates of the SPF-κC composite gels were lower than that of SPI-κC composite gels, demonstrating that κC and protein fibrillation synergistically promote the formation of a dense gel network structure, which slows the riboflavin release. During the simulated intestinal digestion stage, the riboflavin release rates of all composite gel samples rose notably. Two primary mechanisms may account for this phenomenon: (1) The composite gel is affected by the intestinal pH environment. After gastric digestion, the composite gel absorbs water and swells significantly, which partially disrupts the gel network structure and leads to increased riboflavin release [58]. (2) During gastric digestion, the acidic pH environment stabilizes riboflavin and reduces its degradation; conversely, the neutral pH conditions in the intestinal phase facilitate the release of encapsulated riboflavin.

To further elucidate the riboflavin release mechanism from SPI-κC/SPF-κC composite gels in simulated gastrointestinal fluid, the Ritger–Peppas model was employed to process the data and determine the kinetic parameters of the riboflavin release. This model is the most widely used mathematical model for analyzing nonlinear diffusion curves. The release exponent (*n*) bears a direct relation to the riboflavin release mechanism: when *n* ≤ 0.45, the riboflavin release is predominated by simple (Fickian) diffusion; when 0.45 < *n* < 0.89, it is jointly mediated by the combination of diffusion and swelling (non-Fickian diffusion) [59]. Table 1 summarizes the kinetic parameters for riboflavin release from the SPI-κC/SPF-κC composite gels, which was derived via fitting with this model. At κC concentrations below 6 mg/mL, the *n* values for SPI-κC/SPF-κC composite gels were ≤0.45, confirming that riboflavin release from these gels conformed to the Fickian diffusion mechanism. However, at a κC concentration of 8 mg/mL, the *n* value of the SPI-κC/SPF-κC composite gels surpassed 0.45, signifying that high κC concentrations trigger a shift in the riboflavin release mechanism toward non-Fickian diffusion. Notably, the non-Fickian diffusion mechanism facilitates the slow and sustained release of nutrients [60]. Unlike Fickian diffusion driven solely by concentration gradient (prone to premature burst release), non-Fickian diffusion is dominated by gel network swelling and relaxation [61]. The composite gel maintained a compact structure in acidic gastric fluid to minimize riboflavin leakage and degradation, while gradual swelling in neutral intestinal fluid triggered sustained release. This non-Fickian mode achieves uniform release throughout the intestinal phase, which prolongs the riboflavin absorption window [13]. Notably, a clear concentration-dependent trade-off between the protective stability and intestinal release of riboflavin was observed with the increase of κC concentration, which is a core factor for the formulation design of this delivery system. This trade-off effect is attributed to the dual impact of κC on the three-dimensional network structure of the composite gel. On the one hand, the elevated κC concentration enhances the cross-linking density of the gel network via hydrogen bonding and hydrophobic interactions with SPF, which improves the structural stability of the gel, enhances the protective effect on riboflavin under environmental stresses, and reduces the premature leakage of riboflavin during gastric digestion [62,63]. On the other hand, the excessively dense network formed at high κC concentration (8 mg/mL) exerts a strong spatial steric hindrance effect, which not only limits the diffusion of digestive enzymes into the gel matrix to degrade the protein network, but also reduces the equilibrium swelling degree of the gel in neutral intestinal fluid, thereby narrowing the diffusion channels for free riboflavin and ultimately reducing the final release amount of riboflavin [64,65]. In terms of practical application, the κC concentration range of 4–6 mg/mL can balance the protective stability and intestinal release of riboflavin, which is more suitable for conventional food nutritional fortification scenarios; while the 8 mg/mL κC group with non-Fickian diffusion characteristics is more applicable to delivery scenarios requiring long-term targeted sustained release in the posterior intestinal segment.

## 4. Conclusions

In this study, we fabricated tunable soybean protein fibril (SPF)/κ-carrageenan (κC) composite gels, and developed this system as a delivery carrier for riboflavin. The core novelty of this work lies in the systematic elucidation of the synergistic regulation mechanism of protein fibrillation and κC concentration on gel structural stability, riboflavin environmental resistance, and in vitro digestive release behavior, along with the identification of the critical κC concentration threshold that triggers the transition of riboflavin release mechanism. With the increase of κC concentration, both SPI-κC and SPF-κC gels exhibited reduced protein leaching and improved riboflavin retention under multiple environmental stresses, including UV irradiation, thermal treatment, freeze–thaw cycles, pH environment, and storage. Notably, the SPF-κC system showed significantly superior structural stability and riboflavin protective efficacy compared with the SPI-κC counterpart. At 8 mg/mL κC, the riboflavin release mechanism shifted from Fickian to non-Fickian diffusion, which favors the intestinal targeted delivery of riboflavin. Overall, the SPF-κC composite gel system provides a promising solution to the environmental sensitivity and low bioavailability of riboflavin, with broad application potential in food nutritional fortification. However, the static in vitro digestion model used in this study cannot fully replicate the dynamic physiological processes of the human gastrointestinal tract in vivo, such as gastrointestinal peristalsis, continuous secretion and emptying of digestive fluids, and real-time pH changes. Meanwhile, our experimental observations revealed a significant trade-off between κC concentration and riboflavin intestinal bioaccessibility: excessively high κC level (8 mg/mL) enhances the stability of riboflavin, but reduces its final release rate in the intestinal phase. Future research will focus on formulation optimization to balance the stability and bioaccessibility of riboflavin, validation of in vivo bioavailability via animal experiments, and exploration of its application in real food matrices.

## Figures and Tables

**Table 1:** Release kinetic parameters of SPI-κC/SPF-κC riboflavin-loaded composite gels prepared at different concentrations of κC.

Sample	*n*	R^2^
I-4	0.32	0.92
I-6	0.38	0.89
I-8	0.48	0.89
F-2	0.33	0.88
F-4	0.34	0.91
F-6	0.35	0.92
F-8	0.46	0.95

## Data Availability

The original contributions presented in this study are included in this article. Further inquiries can be directed to the corresponding authors.

## References

[1] Pinto J.T., Zempleni J. (2016). Riboflavin. Adv. Nutr..

[2] Suwannasom N., Kao I., Pruss A., Georgieva R., Baeumler H. (2020). Riboflavin: The Health Benefits of a Forgotten Natural Vitamin. Int. J. Mol. Sci..

[3] Ge X., Sun Y., Kong J., Mao M., Yu H., Arora A., Suppavorasatit I., Wang Y. (2022). The thermal resistance and targeting release of zein-sodium alginate binary complexes as a vehicle for the oral delivery of riboflavin. J. Food Sci. Technol..

[4] Nishinari K., Fang Y., Guo S., Phillips G.O. (2014). Soy proteins: A review on composition, aggregation and emulsification. Food Hydrocoll..

[5] O’Sullivan J., Park M., Beevers J. (2016). The effect of ultrasound upon the physicochemical and emulsifying properties of wheat and soy protein isolates. J. Cereal Sci..

[6] Yang B., Dai S., Tang Y., Xu T., Wang J., Zhu W., Xie J., Tong X., Wang H., Jiang L. (2025). Salting-Out Effect Behavior of Protein/λ-Carrageenan Composite Gels Enhanced by Enzymatic Pretreatment: Focusing on Microstructure, Interactions and the Potential for Dysphagia Food. Foods.

[7] Adamcik J., Mezzenga R. (2018). Amyloid Polymorphism in the Protein Folding and Aggregation Energy Landscape. Angew. Chem. Int. Ed..

[8] Yue J., Shu M., Yao X., Chen X., Li D., Yang D., Liu N., Nishinari K., Jiang F. (2022). Fibrillar assembly of whey protein isolate and gum Arabic as iron carrier for food fortification. Food Hydrocoll..

[9] Ji F., Xu J., Liu H., Shao D., Wang C., Zhao Y., Luo S., Zhong X., Zheng Z. (2023). Improved water solubility, antioxidant, and sustained-release properties of curcumin through the complexation with soy protein fibrils. LWT—Food Sci. Technol..

[10] Udo T., Mummaleti G., Mohan A., Singh R.K., Kong F. (2023). Current and emerging applications of carrageenan in the food industry. Food Res. Int..

[11] Alavi F., EmamDjomeh Z., Yarmand M.S., Salami M., Momen S., Moosavi-Movahedi A.A. (2018). Cold gelation of curcumin loaded whey protein aggregates mixed with k-carrageenan: Impact of gel microstructure on the gastrointestinal fate of curcumin. Food Hydrocoll..

[12] Fang Q., Xu T., Su R., Dai S., Wang J., Zhu W., Yang B., Tong X., Wang H., Jiang L. (2025). Composite gel based on κ-carrageenan-soybean isolate protein/soy protein fibrils: Focus on structural differences and gel properties. Int. J. Biol. Macromol..

[13] Cao J., Li L., Yang X. (2025). Enhanced physicochemical properties and riboflavin delivery ability of soy isolate protein/sugar beet pectin composite freeze-dried gels prepared by double crosslinking strategy. Carbohydr. Polym..

[14] Jin B., Zhou X., Li X., Lin W., Chen G., Qiu R. (2016). Self-Assembled Modified Soy Protein/Dextran Nanogel Induced by Ultrasonication as a Delivery Vehicle for Riboflavin. Molecules.

[15] Zhang X., Zhao H., Lu Y., Qi B., Li Y. (2026). Study on the mechanism of riboflavin/UV induced soy protein nanofibrils hydrogel by photo crosslinking. Food Hydrocoll..

[16] Tong X., Cao J., Tian T., Lyu B., Miao L., Lian Z., Cui W., Liu S., Wang H., Jiang L. (2022). Changes in structure, rheological property and antioxidant activity of soy protein isolate fibrils by ultrasound pretreatment and EGCG. Food Hydrocoll..

[17] Wang M., Yang S., Sun N., Zhu T., Lian Z., Dai S., Xu J., Tong X., Wang H., Jiang L. (2024). Soybean isolate protein complexes with different concentrations of inulin by ultrasound treatment: Structural and functional properties. Ultrason. Sonochemistry.

[18] Yang S., Lian Z., Cheng L., Liu X., Dai S., Tong X., Wang H., Jiang L. (2024). Insight into succinylated modified soy protein isolate-sodium alginate emulsion gels: Structural properties, interactions and quercetin release behavior. Food Hydrocoll..

[19] He Z., Ma T., Zhang W., Su E., Cao F., Huang M., Wang Y. (2021). Heat-induced gel formation by whey protein isolate-Lycium barbarum polysaccharides at varying pHs. Food Hydrocoll..

[20] Feng J., Liu S., Sun N., Dong H., Miao L., Wang H., Tong X., Jiang L. (2024). Combining different ionic polysaccharides and pH treatment improved functional properties of soybean protein amyloid fibrils through structural modifications. Food Hydrocoll..

[21] Sun F., Xu J., Wang Z., Cheng T., Wang D., Liu J., Guo Z., Wang Z. (2024). Effect of glycosylation on soy protein isolate–sodium carboxymethyl cellulose conjugates heat-induced gels and their applications as carriers of riboflavin. Food Hydrocoll..

[22] Anwar Z., Noreen A., Usmani M., Akram Z., Ejaz M.A., Sheraz M.A., Ahmed S., Zahid S., Sabir S., Musharraf S.G. (2024). A kinetic study for the estimation of riboflavin sensitized photooxidation of pyridoxine HCl using green UV-visible spectrometric and HPLC methods. RSC Adv..

[23] Yang S., Lian Z., Chi Y., Tang Y., Wang M., Zhang J., Tong X., Wang H. (2026). Insights into ultrasound-modified soy protein isolate-inulin hydrogels: Focus on binding mechanisms, stability, and riboflavin controlled-release behavior. Food Chem..

[24] Clímaco G.N., Gonçalves R.F.S., Fernandes J.-M., Pinheiro A.C., Vicente A.A., Fasolin L.H. (2025). Simultaneous vehiculation of curcumin and riboflavin in bigels produced through WPI cold-set gelation. Food Hydrocoll..

[25] Subramanian P., Nadia J., Paul Singh R., Bornhorst G.M. (2023). Comparison of four digestion protocols on the physical characteristics of gastric digesta from cooked couscous using the Human Gastric Simulator. Food & Function.

[26] Lian Z., Su R., Zhang Q., Tang Y., Yang S., Liu X., Cheng L., Wang H., Jiang L. (2025). Dual modification of soy protein isolate by phlorotannins and enzymatic hydrolysis: Stability and digestive properties. Food Hydrocoll..

[27] Xue C., Lin T.Y., Chang D., Guo Z. (2017). Thioflavin T as an amyloid dye: Fibril quantification, optimal concentration and effect on aggregation. R. Soc. Open Sci..

[28] Biancalana M., Koide S. (2010). Molecular mechanism of Thioflavin-T binding to amyloid fibrils. Biochim. Biophys. Acta (BBA)-Proteins Proteom..

[29] Li T., Wang L., Zhang X., Geng H., Xue W., Chen Z. (2021). Assembly behavior, structural characterization and rheological properties of legume proteins based amyloid fibrils. Food Hydrocoll..

[30] Wang N., Ma Z., Ma L., Zhang Y., Zhang K., Ban Q., Wang X. (2023). Synergistic modification of structural and functional characteristics of whey protein isolate by soybean isoflavones non-covalent binding and succinylation treatment: A focus on emulsion stability. Food Hydrocoll..

[31] Wang T., Wang N., Yu Y., Yu D., Xu S., Wang L. (2023). Study of soybean protein isolate-tannic acid non-covalent complexes by multi-spectroscopic analysis, molecular docking, and interfacial adsorption kinetics. Food Hydrocolloids.

[32] Lian Z., Yang S., Liu X., Zhang Q., Tan X., Zhao K., Jiang L., Wang H. (2025). Soy glycinin-carboxymethyl cellulose “chain bead-like” nanocomplexes: Focus on formation mechanism, functional characteristics and stability properties. Food Hydrocoll..

[33] Hu C., Xiong Z., Xiong H., Chen L., Zhang Z., Li Q. (2020). The formation mechanism and thermodynamic properties of potato protein isolate-chitosan complex under dynamic high-pressure microfluidization (DHPM) treatment. Int. J. Biol. Macromol..

[34] Joshi M., Aldred P., Panozzo J.F., Kasapis S., Adhikari B. (2014). Rheological and microstructural characteristics of lentil starch-lentil protein composite pastes and gels. FOOD Hydrocoll..

[35] Fitzpatrick A.W., Knowles T.P.J., Waudby C.A., Vendruscolo M., Dobson C.M. (2011). Inversion of the Balance between Hydrophobic and Hydrogen Bonding Interactions in Protein Folding and Aggregation. PLoS Comput. Biol..

[36] Zhao Y., Wang D., Xu J., Tu D., Zhuang W., Tian Y. (2024). Effect of polysaccharide concentration on heat-induced Tremella fuciformis polysaccharide-soy protein isolation gels: Gel properties and interactions. Int. J. Biol. Macromol..

[37] Ge G., Han Y., Zheng J., Zhao M., Sun W. (2020). Physicochemical characteristics and gel-forming properties of myofibrillar protein in an oxidative system affected by partial substitution of NaCl with KCl, MgCl_2_ or CaCl_2_. Food Chem..

[38] Li H., Cao Y., Wang L., Wang F., Xiong L., Shen X., Song H. (2025). Pickering high internal phase emulsions stabilized by soy protein isolate/κ-carrageenan complex for enhanced stability, bioavailability, and absorption mechanisms of nobiletin. Carbohydr. Polym..

[39] Li J., Wang Z., Yang B., Peng X., Liu S., Sun N., Su R., Tong X., Wang H., Jiang L. (2026). Time and pH regulation of the morphology and structure evolution of soybean protein fibrils: Focusing on foaming mechanism and application. Food Hydrocoll..

[40] Jiang L., Ren Y., Xiao Y., Liu S., Zhang J., Yu Q., Chen Y., Xie J. (2020). Effects of Mesona chinensis polysaccharide on the thermostability, gelling properties, and molecular forces of whey protein isolate gels. Carbohydr. Polym..

[41] Dai L., Nan J., Tu X., He L., Wei B., Xu C., Xu Y., Li S., Wang H., Zhang J. (2019). Improved thermostability and cytocompatibility of bacterial cellulose/collagen composite by collagen fibrillogenesis. Cellulose.

[42] Liu R., Yan X., Liu R., Wu Q., Gao Y., Muhindo E.M., Zhi Z., Wu T., Sui W., Zhang M. (2024). Lima bean (Phaseolus lunatus Linn.) protein isolate as a promising plant protein mixed with xanthan gum for stabilizing oil-in-water emulsions. J. Sci. Food Agric..

[43] Li W., Nian Y., Huang Y., Zeng X., Chen Q., Hu B. (2019). High Loading Contents, Distribution and Stability of β-Carotene Encapsulated in High Internal Phase Emulsions. Food Hydrocoll..

[44] Cao J., Tong X., Cao X., Peng Z., Zheng L., Dai J., Zhang X., Cheng J., Wang H., Jiang L. (2024). Effect of pH on the soybean whey protein-gum arabic emulsion delivery systems for curcumin: Emulsifying, stability, and digestive properties. Food Chem..

[45] Dai C., Han S., Ma C., McClements D.J., Xu D., Chen S., Liu X., Liu F. (2024). High internal phase emulsions stabilized by pea protein isolate-EGCG-Fe3+ complexes: Encapsulation of β-carotene. Food Hydrocoll..

[46] Zhou X., Lin H., Wu C.-Y., Sablani S.S., Tang J. (2025). A new chemical marker method for determining heating patterns in microwave pasteurization. J. Food Eng..

[47] Zhai Y., Xing J., Luo X., Zhang H., Yang K., Shao X., Chen K., Li Y. (2021). Effects of Pectin on the Physicochemical Properties and Freeze-Thaw Stability of Waxy Rice Starch. Foods.

[48] Marquez A.L., Salvatore G.N., Otero R.G., Wagner J.R., Palazolo G.G. (2015). Impact of freeze-thaw treatment on the stability of calcium-fortified soy beverages. LWT—Food Sci. Technol..

[49] Xu Y., Tao Y., Shivkumar S. (2016). Effect of freeze-thaw treatment on the structure and texture of soft and firm tofu. J. Food Eng..

[50] Cui Q., Liu J., Wang G., Zhang A., Wang X., Zhao X.-h. (2022). Effect of freeze-thaw treatment on the structure and texture of soy protein-dextran conjugate gels crosslinked by transglutaminase. LWT—Food Sci. Technol..

[51] Levin P.P., Tatikolov A.S., Panova I.G., Sul’timova N.B. (2010). A laser photolysis study of the decay kinetics of the triplet states and radicals of flavins in the bovine eye lens. High Energy Chem..

[52] Xu Q., Qi B., Han L., Wang D., Zhang S., Jiang L., Xie F., Li Y. (2021). Study on the gel properties, interactions, and pH stability of pea protein isolate emulsion gels as influenced by inulin. LWT—Food Sci. Technol..

[53] Zhao K., Hao Y., Guo X., Chang Y., Shen X. (2025). Development and characterization of quinoa protein-fucoidan emulsion gels with excellent rheological properties for 3D printing and curcumin encapsulation stability. Food Chem..

[54] Huang X., Liu B., Li Y., Huang D., Zhu S. (2024). Influence mechanism of components and characteristics on structural and oxidative stability of emulsion gel. Food Hydrocoll..

[55] Cao J., Zhao H., Peng Z., Yang B., Xu H., Cheng J., Wang H. (2025). The effects of non-covalent interaction between rice glutelin and gum arabic on digestibility and stability of perilla oil emulsion. Food Chem..

[56] Liu D., Janssen A.E.M., Smeets P.A.M., Stieger M. (2024). Interplay between microstructure, mechanical properties, macrostructure breakdown and in vitro gastric digestion of whey protein gels. Food Hydrocoll..

[57] Li X., Zhang X., Li J., Zhang W., Gong D., Li F. (2023). Assembly of perilla seed protein isolate-pectin nanocomplex to deliver curcumin: Properties, characterization, molecular interactions and antioxidant activity. Food Biosci..

[58] Basak S., Singhal R.S. (2024). Inclusion of konjac glucomannan in pea protein hydrogels improved the rheological and in vitro release properties of the composite hydrogels. Int. J. Biol. Macromol..

[59] Misra G.P., Singh R.S.J., Aleman T.S., Jacobson S.G., Gardner T.W., Lowe T.L. (2009). Subconjunctivally implantable hydrogels with degradable and thermoresponsive properties for sustained release of insulin to the retina. Biomaterials.

[60] Cheng T., Wang Z., Sun F., Liu H., Liu J., Guo Z., Zhou L. (2024). Gel properties of rice proteins-pectin composite and the delivery potential for curcumin: Based on different concentrations and the degree of esterification of pectin. Food Hydrocoll..

[61] Saidi M., Dabbaghi A., Rahmani S. (2020). Swelling and drug delivery kinetics of click-synthesized hydrogels based on various combinations of PEG and star-shaped PCL: Influence of network parameters on swelling and release behavior. Polym. Bull..

[62] Yang C., Wang Y., Chen L. (2017). Fabrication, characterization and controlled release properties of oat protein gels with percolating structure induced by cold gelation. Food Hydrocoll..

[63] Zhu Q., Han K., Wei W., Zhang L., Gao J., Wu T., Zhao T., Chen H., Zhang M. (2022). Rational design cold-set interpenetrating network hydrogel based on wheat bran arabinoxylans and pea protein isolates for regulating the release of riboflavin in simulated digestion. Int. J. Biol. Macromol..

[64] Li R.-X., Yuan J.-L., Ding C.-S., Kang X. (2022). Bovine serum albumin cold-set emulsion gel mediated by transglutaminase/glucono-δ-lactone coupling precursors: Fabrication, characteristics and embedding efficiency of hydrophobic bioactive components. LWT—Food Sci. Technol..

[65] Wu R., Huang L., Chen L., Li Y., He R. (2025). Influence of gelation methods on the physicochemical properties and release behavior of whey protein isolate/κ-carrageenan gels. Food Res. Int..

